# Krüppel-like factor 5 -mediated Sirtuin6 promotes osteogenic differentiation and inhibits inflammatory injury of lipopolysaccharide-induced periodontal membrane stem cells by inhibiting nuclear factor kappa-B pathway

**DOI:** 10.1080/21655979.2022.2036915

**Published:** 2022-03-07

**Authors:** Chanxiu Li, Feng Xiao, Yunsheng Wen, Jian Wu, Nannan Huang

**Affiliations:** Department of Stomatology, The Third Affiliated Hospital of Sun Yat-sen University, Guangzhou, China

**Keywords:** KLF5, SIRT6, osteogenic differentiation, inflammatory injury, periodontal membrane stem cells, NF-κB pathway

## Abstract

Periodontitis is a chronic infectious disease that causes inflammation and immune response and has an ultimate impact on the health of the whole body. Sirtuin6 (SIRT6) and Krüppel-like factor 5 (KLF5) have been reported to regulate the inflammatory response and play an important role in the development of periodontitis. LPS was adopted to induce periodontal ligament stem cells (PDLSCs) to construct a periodontitis cell model. SIRT6 expression was assayed through RT-qPCR and Western blot. Subsequently, after SIRT6 was overexpressed, CCK8 was to appraise cell viability. ELISA analysis was used to estimate inflammatory response. ALP staining, ARS staining, and Western blot were used to detect osteogenic differentiation. The JASPAR website then predicts the binding of transcription factor KLF5 to SIRT6 promoter. The interaction between KLF5 and SIRT6 was verified by a luciferase reporter and ChIP assays. Additionally, the osteogenic differentiation and inflammation in LPS-induced PDLSCs transfected with Ov-SIRT6 and si-KIF5 were also explored. Finally, the protein levels of the nuclear factor kappa-B (NF-κB) pathway-related factors were detected by Western blot to further explore the mechanism. There was a marked decrease in SIRT6 expression in LPS-induced PDLSCs. SITR6 overexpression prevented LPS-induced cell viability loss and inflammation, while promoting osteogenic differentiation. In addition, KLF5 could transcriptionally activate SIRT6. Further, KLF5 knockdown reversed the impacts of SIRT6 on the proliferation, inflammation, and osteogenic differentiation of LPS-induced PDLSCs via mediating NF-κB pathway. Overall, KLF5-mediated SIRT6 promoted the viability and osteogenic differentiation, while inhibiting the inflammatory response of LPS-induced PDLSCs by inhibiting NF-κB pathway.

## GRAPHICAL ABSTRACT

**Figure:**
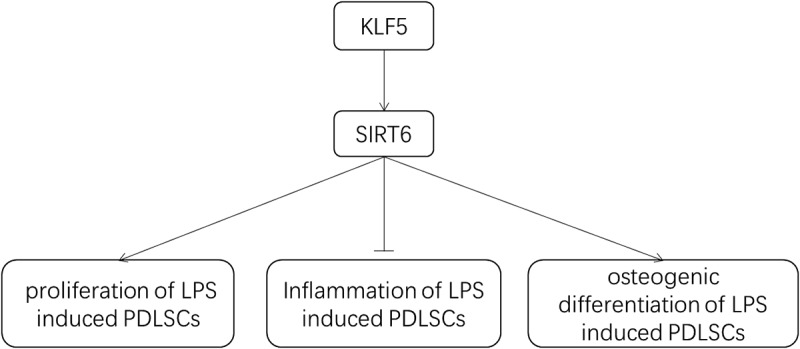

## Introduction

Periodontitis is an oral disease that is most frequently found in humans and is the main cause of adult tooth loss. It seriously affects the function of the oral and maxillary system [1]. Periodontitis is usually caused by infection with Gram-negative anaerobe from periodontal supporting tissues [2]. Periodontitis occurs in up to 80% of the adults, 20% of whom have a more severe condition. In addition, as a long-term persistent source of infection, periodontitis can also trigger chronic inflammation and immune response in the human body, thereby having an important impact on systemic health [3]. Hence, it is essential to delve into the pathogenesis of periodontitis to provide novel insights for periodontitis treatment.

Periodontal ligament stem cells (PDLSCs) are a type of oral adult stem cells whose heterogeneity is closely related to the regeneration and repair of periodontal tissues [4]. PDLSCs exist at different stages of differentiation with non-directional differentiation potential. PDLSCs can also proliferate and produce fibroblasts, osteoblasts, and cementoblasts [5]. A large number of animal experiments and clinical case reports showed that great improvements have been achieved in repairing periodontal tissue defects with transplantation of PDLSCs [6–8]. Therefore, exploring the mechanism underlying the differentiation of PDLSCs is of great significance to get a better understanding of periodontitis therapy.

Silent mating-type information regulation 2 homolog-6 (SIRT6) possesses many biological functions, such as maintaining genome stability [9], regulating the metabolism of both glucose and lipid [10], regulating inflammation, apoptosis, and cell senescence [11]. Osteoblasts differ in their morphological basis, origin, differentiation, and cell regulation. SIRT6 expression was discovered to increase in the early stage of osteoblast differentiation and decrease in the late stage. Overexpression of SIRT6 suppressed the differentiation and mineralization of osteoblasts. Huang et al. proposed that the down-regulation of SIRT6 promoted cementum formation [12]. On the contrary, it was reported that SIRT6 deficiency in osteoblast lineage cells could cause osteoporosis by activating osteoclasts [13]. However, there have been few studies of the effect of SIRT6 on PDLSCswithi periodontitis.

Possible binding of the transcription factor KLF5 to SIRT6 promoter was predicted by the JASPAR website (http://jaspar.genereg.net) [14]. A previous study showed that miR-143-3p inhibited osteogenic differentiation of human PDLSCs by targeting KLF5 and inactivating Wnt/β-catenin pathways [15]. DNMT3B-mediated hypermethylation of the KLF5 promoter induced by oxidative stress impaired osteogenesis by reducing its interaction with β-catenin [16]. IL1β-, flagellin- and poly(I:C)-stimulated NF-κB transcriptional activity was significantly suppressed in KLF5-knockout myometrial cells [17]. Therefore, it has been hypothesized that KLF5 might play a role in osteoblast differentiation and inflammation of PDLSCs through transcriptional regulation of SIRT6.

Therefore, in this study, we aimed to examine the roles of KLF5 and SIRT6 in LPS-induced osteogenic differentiation and inflammatory response of PDLSCs so as to explore the pathogenesis of periodontitis.

## Materials and methods

### Cell culture

The periodontal ligament stem cells (PDLSCs) were provided by iCell Bioscience Inc (HUM-iCELL-m002). The DMEM (Gibco) with 10% fetal bovine serum (Gibco) was used to culture PDLSCs and was placed in an incubator at 37°C with 5% CO_2。_PDLSCs were induced with lipopolysaccharide (LPS) at a dose of 1 μg/mL for 12, 24, and 48 h.

### Database

JASPAR website (http://jaspar.genereg.net) was used to predict the binding between KLF5 and SIRT6 [14].

### Reverse transcription quantitative real-time PCR (RT-qPCR)

When the cells were fused to 80%, Trizol Reagent was used to isolate the total RNA, and then the cells were treated with DNase. cDNA was synthesized using Superscript III (Life Technologies) cDNA kit with the Random Hexamer primer. SYBR Green Master Mix (BioRad) was used to perform PCR on the BioRad CFX Connect Real Time machine using 30 ng of cDNA per reaction with 4x reactions. Denaturation, annealing, and extension were performed under the following conditions: 95^°^C for 30 sec, 95^°^C for 5 sec for a total of 45 cycles, and 60^°^C for 20 sec. Relative gene expression was analyzed by the 2^−ΔΔCt^ method [18] and standardized by the GAPDH level. The primers were listed as below:

SIRT6 F: 5’-GCAGTCTTCCAGTGTGGTGT- 3’, SIRT6 R: 5’-GATCTGCAGCGATGTACCCA- 3’.

Osteocalcin (OCN) F: 5’-GCAGTCTTCCAGTGTGGTGT-3’, OCN R: 5’-GATCTGCAGCGATGTACCCA- 3’.

RUNX2 F: 5’- CGCCTCACAAACAACCACAG- 3’, RUNX2 R: 5’- TCACTGTGCTGAAGAGGCTG- 3’.

BMP2 F: 5’- ACTCGAAATTCCCCGTGACC- 3’, BMP2 R: 5’- CCACTTCCACCACGAATCCA- 3’.

KLF5 F: 5’- ACGCTTGGCCTATAACTTGGTT- 3’, KLF5 R: 5’- TGATGTGTGTTACGCACGGT- 3’.

GAPDH F: 5’-AATGGGCAGCCGTTAGGAAA- 3’, GAPDH R: 5’-GCGCCCAATACGACCAAATC- 3’.

### Western blot

Cell lysis of PDLSCs was completed in RIPA buffer (Beyotime), followed by protein concentration measurements using a BCA kit (Pierce). A total of 10% SDS-PAGE was employed to isolate the proteins, which were then carried out on PVDF membranes. Blocking of the membrane was done with 5% skim milk before overnight incubation with primary antibodies (1:1000, Abcam) at 4°C. Following washing by PBST the next day, the incubation of membranes was carried out at room temperature with the secondary antibody (1:5,000; Abcam) labeled with horseradish peroxidase for 1 h. The visualization of protein bands was undertaken with the application of enhanced chemiluminescence reagents (Bio-Rad, CA, USA). The analysis of protein bands was conducted with the use of ImageJ 1.52 k software (Version146, National Institutes of Health).

### Cell Counting Kit-8 (CCK8)

After transfection, the cells were exposed to LPS for 48 h. 10 μl of CCK-8 solution (Dojindo Molecular Technologies, Inc., Kumamoto, Japan) was then co-incubated with these cells for another 4 h at 37°C. The optical density (450 nm) was measured with a microplate reader (Bio-Rad, Gaithersburg, MD, USA).

### Cell transfection

SIRT6 (Ov-SIRT6) and KLF5 (Ov-KLF5) overexpression and empty control plasmids were originally supplied by Thermo Fisher Scientific. The small interfering RNAs (siRNAs) against KLF5-1 and KLF5-2 (si-KLF5-1/2) and the control siRNA were also provided by Thermo Fisher Scientific. PDLSCs were grown in 6-well plates (1.0 × 10^5^ cells/well) for 12 h prior to transfection. Cells at 80% confluence were transiently transfected with a total of 20 μM transfection content as per the manufacturer’s instructions utilizing Lipofectamine 3000 (Invitrogen/Thermo Fisher Scientific) for 48 h.si-NC: CTTACGCTGAGTACTTCGA, si-KLF5: GAUUACCCUGGUUGCACA,

### ELISA

The levels of TNF-α, IL-6, and IL-1β were determined following the instructions of the manufacturer through the use of ELISA kits (Nanjing Jiancheng, Nanjing, China).

### Alkaline phosphatase (ALP) staining

PDLSCs were plated in 6-well plates (2.0 × 10^5^ cells/well) to achieve 80% confluence and changed osteogenic-inducing medium using the StemPro osteogenesis differentiation kit (Invitrogen, USA). The cells were then exposed to a medium for the purpose of osteogenic induction. Following successful induction, PDLSCs were covered with appropriate amount of BCIP/NBT dyeing solution at room temperature in the dark for 20 min and observed under a light microscope. A 400 μl of cell lysate were added into the cells, and then the supernatant was collected after centrifugation at 1000 *g* for 10 min. At last, 100 μl of reaction termination solution were added to abort the reaction. After being cultured for 7 days, the procedures were conducted strictly following the recommendations of the ALP test kit (Thermo Fisher Scientific Inc.). The absorbance (405 nm) was determined using a microplate reader (BioTek, Winooski, VT, USA) [19].

### Alizarin red S (ARS) staining

PDLSCs were plated in 6-well plates (2.0 × 10^5^ cells/well) to achieve 80% confluence and changed osteogenic-inducing medium using the StemPro osteogenesis differentiation kit (Invitrogen, USA). The differentiated PDLSCs were fixed in 4% formaldehyde for 15 min at room temperature, washed with PBS and stained with 2% alizarin red solution (Beyotime Biotechnology, Shanghai, China) at room temperature. After washing the cells using distilled water, the calcium nodules were observed with the help of a microscope. Calcium nodules were solubilized with 10% cetylpyridinium chloride (CPC) for 30 min to quantify the matrix mineralization. Absorbance of calcium concentrations was estimated at 562 nm [20].

### Luciferase reporter assay

The SIRT6 reporter, which contains 1 kb of the SIRT6 promoter, was created using RT-qPCR as aforementioned and cloned into the pGL3 plasmid (Shanghai GenePharma Co., Ltd). The wild type (WT) and mutant type (MUT) of SIRT6 promoter were co-transfected with Ov-KLF5, Ov-NC, and pRL-TK (internal reference plasmids expressing renilla luciferase) into PDLSCs. The cells were lysed according to the protocol of TransDetect Double-Luciferase Reporter Assay Kit (FR201-01, TransGen Biotech, Beijing, China) following 48 h of transfection, and the supernatant was then collected. The luciferase activity was detected by Dual-luciferase reporter assay system (E1910, Promega). Firefly luciferase/renilla luciferase was adopted as the relative luciferase activity [20].

### Chromatin immunoprecipitation (ChIP) assay

ChIP was performed on the basis of the specifications of the Magna ChIP HiSens Kit (Millipore, Darmstadt, Germany) [21]. Briefly, following cross-linking of cells by 1% formaldehyde, cells were sonicated on ice to create 200–500 bp fragments. Stained chromatin was cultured overnight with anti-KLF5 antibody (1:50, Abcam) or IgG (Abcam) as an isotype control. The precipitated chromatin DNA was recovered and subjected to RT-qPCR analysis.

### Statistical analysis

All data obtained from the experiments were displayed in the form of mean and standard deviation (means ± SD) and processed applying SPSS 16.0 software developed by SPSS company (Chicago, Illinois, USA). A one-way analysis of variance (ANOVA) followed by Tukey’s post-hoc test was used to make multiple comparisons among groups. Student’s t-test was employed to analyze data between the two groups. It was deemed to be statistically significant at *P* < 0.05. Each experiment was conducted at least three times.

## Results

### Overexpression of SIRT6 increased the viability of LPS-induced PDLSCs

**Figure 1:**
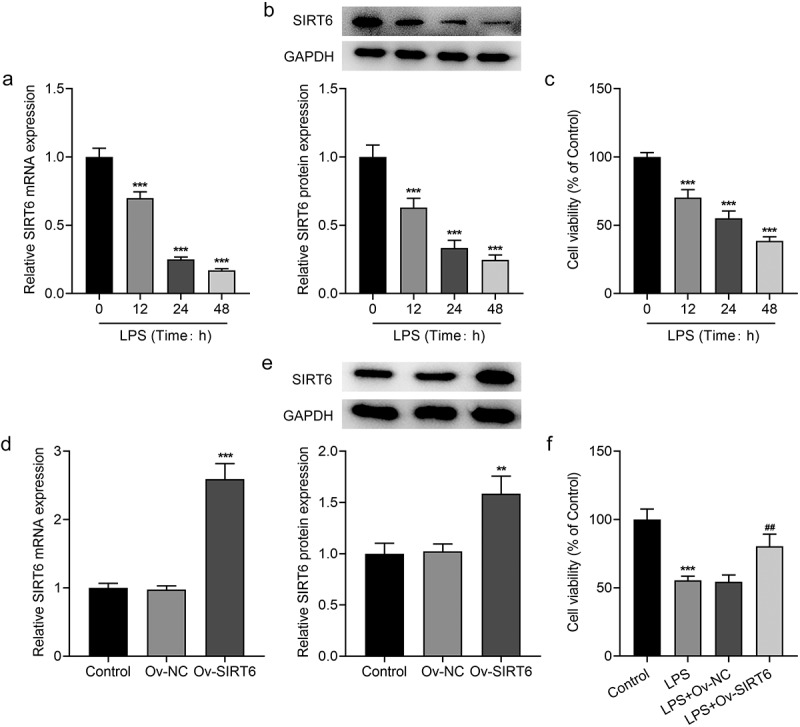
Overexpression of SIRT6 increased the viability of LPS-induced PDLSCs.

As displayed in Figure 1(a,b), SIRT6 expression in LPS-induced PDLSCs was decreased in a time-dependent manner. The alternation of cell viability after LPS induction was detected by CCK-8. It was found that LPS treatment markedly suppressed the viability of PDLSCs and cell viability was reduced gradually with increasing induction time (Figure 1(c)). 24 h of LPS induction time was confirmed in the following experiments. Subsequently, we transfected Ov-SIRT6 into PDLSCs, and RT-qPCR and Western blot analysis verified the excellent overexpression efficiency of SIRT6 (Figure 1(d,e)). Moreover, CCK-8 results showed that cell viability in LPS+Ov-SIRT6 group was significantly increased compared with LPS+Ov-NC group (Figure 1(f)).

a. RT-qPCR was employed to detect SIRT6 mRNA level in PDLSCs after LPS induction for 12, 24, and 48 h. n = 5. b. Western blot was employed to assess SIRT6 protein level in PDLSCs after LPS induction for 12, 24, and 48 h. n = 3. c. CCK-8 assay was used to measure cell viability after LPS induction for 12, 24, and 48 h. n = 5. ***p < 0.001 vs LPS (Time: 0 h). d. RT-qPCR was employed to detect SIRT6 mRNA level after plasmid transfection for 48 h. n = 5. e. Western blot was applied to examine SIRT6 protein level after plasmid transfection for 48 h. n = 3. **p < 0.01, ***p < 0.001 vs Ov-NC. f. CCK-8 was adopted to estimate the cell viability after plasmid transfection. ***p < 0.001 vs Control, ^##^p < 0.01 vs LPS+Ov-NC.

### Overexpression of SIRT6 alleviated inflammatory response of LPS-induced PDLSCs

**Figure 2:**
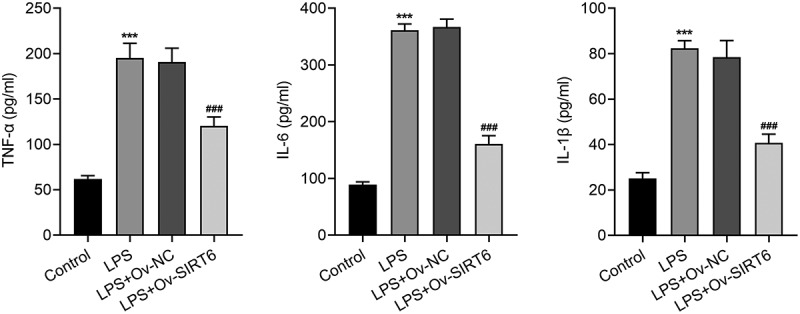
Overexpression of SIRT6 attenuated LPS-induced inflammation in PDLSCs. ELISA assay was utilized to examine the levels of TNF-α, IL-6 and IL-1β afte*r plasmid* transfection in LPS induced PDLSCs. n = 5. ***p < 0.001 vs Control, ^###^p < 0.001 vs LPS+Ov-NC.

The inflammatory response of LPS-induced PDLSCs was subjected to evaluation by ELISA. It was evidently observed that TNF-α, IL-6, and IL-1β expression was increased after LPS induction (vs Control), while the levels of TNF-α, IL-6, and IL-1β in LPS-induced PDLSCs transfected with Ov-SIRT6 were markedly inhibited in comparison with LPS+Ov-NC (Figure 2).

### Overexpression of SIRT6 promoted osteogenic differentiation of LPS-induced PDLSCs

**Figure 3:**
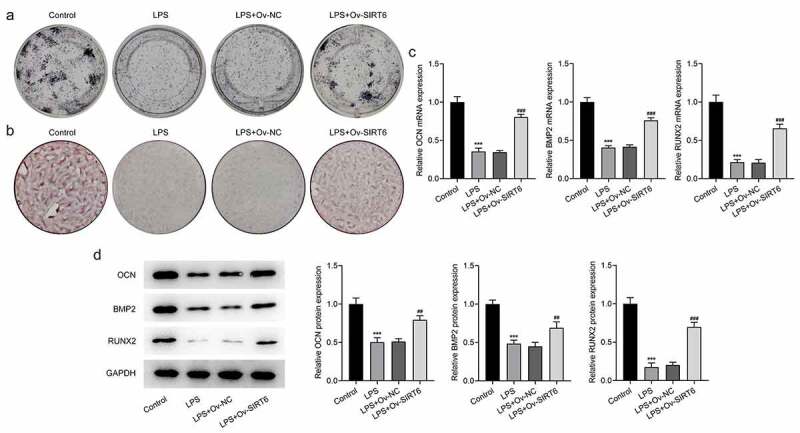
Overexpression of SIRT6 promoted osteogenic differentiation of LPS-induced PDLSCs.

The results of ALP staining suggested that the osteogenic differentiation ability of cells in the LPS group was apparently diminished (vs Control). Compared with LPS+Ov-NC group, there was an increase in the osteogenic ability of cells in the LPS+Ov-SIRT6 group (Figure 3(a)). Meanwhile, ARS staining revealed that there were fewer mineralized nodules in the LPS group (vs Control). Compared with LPS+Ov-NC group, the number of mineralized nodules was increased in LPS+Ov-SIRT6 group (Figure 3(b)). Furthermore, it could be seen that the expression of osteogenic markers including OCN, RUNX2, and BMP2 was decreased in LPS group (vs Control). Compared with LPS+Ov-NC group, OCN, RUNX2, and BMP2 expressions were increased in LPS+Ov-SIRT6 group (Figure 3(c,d)).

a. The osteogenic differentiation ability of LPS-induced PDLSCs was determined with the application of ALP staining. n = 3. b. ARS staining was applied to determine the formation of mineralized nodules. n = 3. c. RT-qPCR was utilized to assess the mRNA levels of OCN, BMP2, and RUNX2 after plasmid transfection in LPS-induced PDLSCs. n = 5.d. The protein levels of OCN, BMP2, and RUNX2 were examined with the aid of Western blot after plasmid transfection in LPS-induced PDLSCs. n = 3. ***p < 0.001 vs Control, ^##^p < 0.01, ^###^p < 0.001 vs LPS+Ov-NC.

### Transcription factor KLF5 stimulated the up-regulation of SIRT6

**Figure 4:**
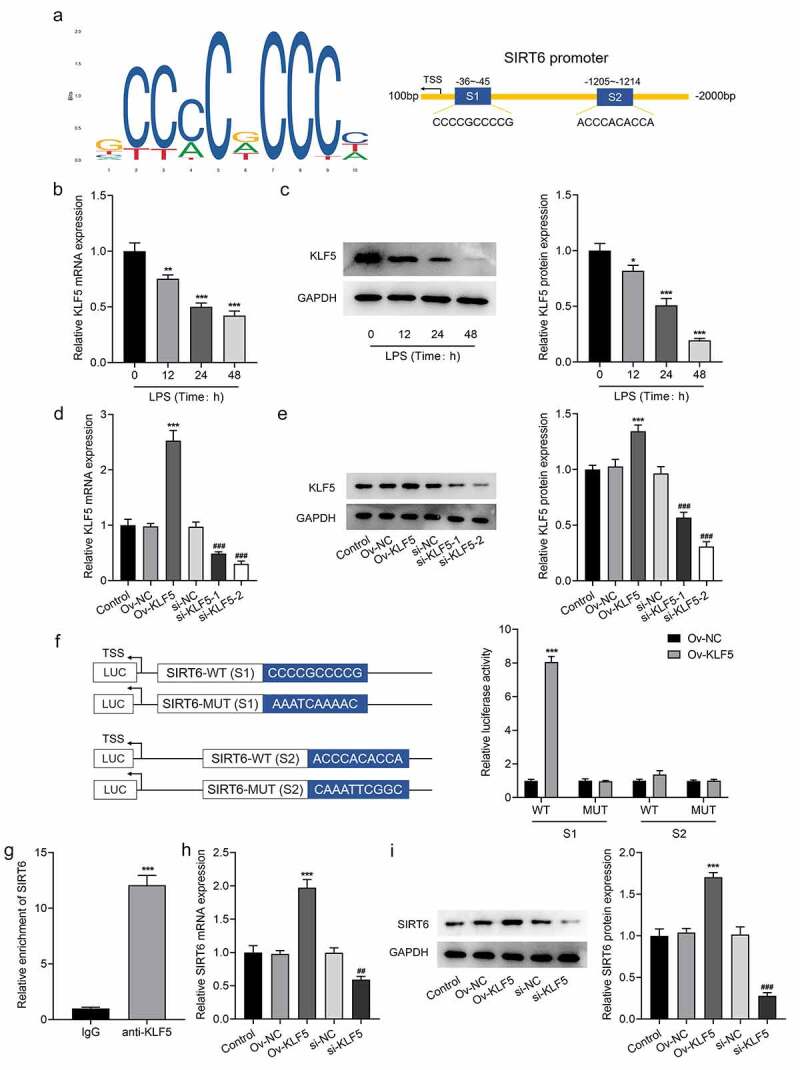
The transcription factor KLF5 stimulated the up-regulation of SIRT6.

The JASPAR website predicts that transcription factors KLF5 and the promoter of SIRT6 had potential-binding sites (Figure 4(a)). It was noticed that there was a marked decrease in KLF5 expression after LPS induction (Figure 4(b,c)). Subsequently, KLF5 expression was determined for the LPS-induced PDLSCs transfected with Ov-KLF5 or si-KLF5 (Figure 4(d,e)). The results imply that there is a noticeable decline in the expression of KLF5 in si-KLF5-2 group than in si-KLF5-1 group, so we selected si-KLF5-2 for follow-up experiments. Also, KLF5 expression was elevated after transfection of Ov-KLF5. Luciferase was then used to verify the binding between KLF5 and SIRT6 promoter. It was found that the overexpression of KLF5 significantly increased the luciferase activity at the S1 site in the WT of SIRT6 promoter compared with the OV-NC (Figure 4(f)), which indicated that KLF5 had a binding site with SIRT6 promoter at the S1 site. The ChIP assay also verified the binding ability of KLF5 to SIRT6 promoter (Figure 4(g)). In addition, SIRT6 expression was elevated in Ov-KLF5 group compared to the Ov-NC group. Further, SIRT6 in si-KLF5 group was remarkably decreased relative to the si-NC group (Figure 4(h,i)). These results suggested that KLF5 can transcriptionally activate SIRT6.

a. JASPAR website was used to predict the binding between KLF5 and SIRT6 promoter. b. RT-qPCR was applied to check the expression of KLF5 in PDLSCs after LPS induction for 12 h, 24 h and 48 h. n = 5. c. Western blot was employed to assay the expression of KLF5 in PDLSCs after LPS induction for 12 h, 24 h and 48 h. n = 3. *p < 0.05, **p < 0.01, ***p < 0.001 vs LPS (Time: 0 h). d. RT-qPCR was used to measure the expression of KLF5 after plasmid transfection. n = 5. e. Western blot was utilized to test the expression of KLF5 after plasmid transfection. n = 3. ***p < 0.001 vs Ov-NC,^###^p < 0.001 vs si-NC. f. The luciferase activity of SIRT6 promoter was examined through luciferase reporter assay. ***p < 0.001 vs Ov-NC. g. ChIP assay was employed to measure the binding ability of KLF5 and SIRT6 promoter. n = 3. ***p < 0.001 vs IgG. h. RT-qPCR was examined to test the expression of SIRT6 after KLF5 was overexpressed or silenced. n = 5. i. The expression of SIRT6 after KLF5 was overexpressed or silenced with the application of Western blot. n = 5. ***p < 0.001 vs Ov-NC,^##^p < 0.01, ^###^p < 0.001 vs si-NC.

### Interference with KLF5 reversed the impacts of SIRT6 on proliferation, inflammation, and osteogenic differentiation of LPS-induced PDLSCs

**Figure 5:**
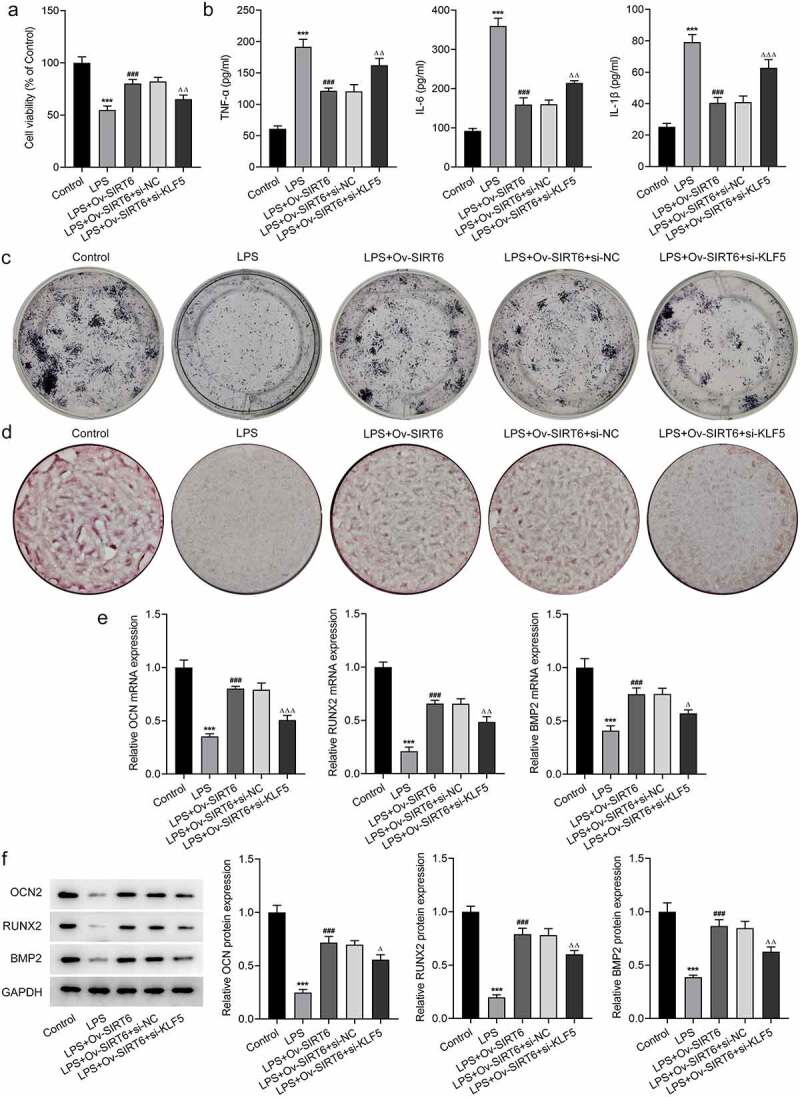
Interference with KLF5 reversed the impacts of SIRT6 on proliferation, inflammation and osteogenic differentiation of LPS-induced PDLSCs.

For the further investigation of the mechanism of KLF5/SIRT6, LSP-induced PDLSCs were co-transfected with Ov-SITR6 and si-KLF5. Compared with LPS+Ov-SIRT6+ si-NC group, PDLSCs exhibited lower viability in LPS + Ov-SIRT6+ si-KLF5 group (Figure 5(a)). Additionally, TNF-α, IL-6, and IL-1β expression were elevated in LPS+Ov-SIRT6+ si-KLF5 group compared with LPS+Ov-SIRT6+ si-NC group (Figure 5(b)). ALP staining showed that inhibition of KLF5 expression reversed the strengthened osteogenic ability of LPS-induced cells caused by SIRT6 overexpression (Figure 5(c)). Besides, by contrast with LPS+Ov-SIRT6+ si-NC group, the number of mineralized nodules in LPS+Ov-SIRT6+ si-KLF5 group was reduced (Figure 5(d)). Then, compared with LPS+Ov-SIRT6+ si-NC group, the expression of OCN, RUNX2, and BMP2 was significantly decreased in LPS+Ov-SIRT6+ si-KLF5 group (Figure 5(e,f)).

a. The viability of PDLSCs after plasmid transfection was examined applying CCK-8. n = 5. b. The levels of TNF-α, IL-6, and IL-1β in PDLSCs after plasmid transfection, were estimated by means of ELISA. n = 5. c. The osteogenic differentiation ability was assessed with the aid of ALP staining. n = 3. d. ARS staining was adopted to determine the formation of mineralized nodules. n = 3. e. The mRNA levels of OCN, BMP2, and RUNX2 were assayed with the help of RT-qPCR. n = 5. f. Western blot was used to determine the protein levels of OCN, BMP2, and RUNX2. n = 3. ***p < 0.001 vs Control, ^###^p < 0.001 vs LPS, ^Δ^p<0.05, ^ΔΔ^p<0.01, ^ΔΔΔ^p<0.001 vs LPS+Ov-SIRT6+ si-NC.

### KLF5-mediated SIRT6 inhibited the NF-κB pathway

**Figure 6:**
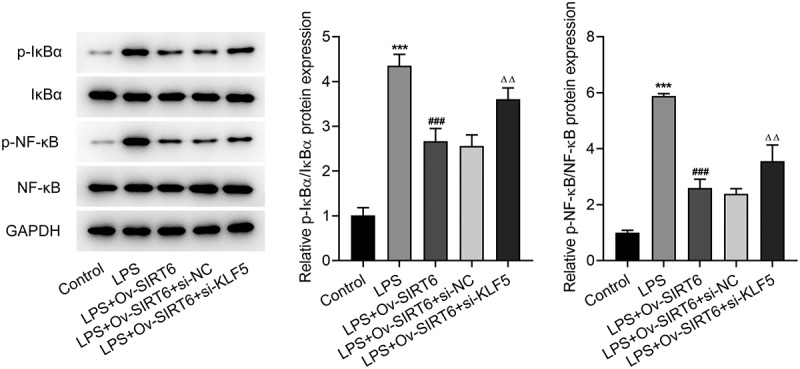
KLF5-mediated SIRT6 inhibited the NF-κB pathway.

It is well documented that the dysregulation of NF-κB signaling is responsible for the progression of human diseases [22]. To determine whether NF-κB pathway was involved in periodontitis, the protein levels of NF-κB pathway-related factors were analyzed by Western blot. Accordingly, the expression of p-I κBα and p-NF -κB was significantly increased after LPS induction and decreased after overexpression of SIRT6 compared with the LPS group. Compared with LPS+Ov-SIRT6+ si-NC group, the expression of p-I κBα and p-NF -κB was reversed in LPS+Ov-SIRT6+ si-KLF5 group (Figure 6). Therefore, it was preliminarily concluded that KLF5-mediated SIRT6 inhibited NF-κB pathway.

The expression of NF-κB pathway-related proteins was assayed with the adoption of Western blot after plasmid transfection in LPS-induced PDLSCs. n = 3. ***p < 0.001 vs Control, ^###^p < 0.001 vs LPS, ^Δ^p<0.05, ^ΔΔ^p<0.01 vs LPS + Ov-SIRT6 + si-NC.

### Discussion

The inflammatory microenvironment in which periodontal tissues of periodontitis patients exist may lead to the destruction rather than reconstruction of periodontal tissues, causing loss of periodontal supporting tissues [23]. As the basis of tissue engineering, stem cells are possible to regenerate periodontal tissues by reconstructing the tissue defects [24]. However, inflammatory induction can reduce osteogenic differentiation of PDLSCs, thus affecting the effect of PDLSCs on periodontal tissue regeneration and differentiation [25]. In addition, study has shown that LPS-induced inflammation in PDLSCs affects the proliferation, oxidative stress, and osteogenic differentiation [26]. Therefore, LPS-induced PDLSCs were used to simulate periodontitis in vitro in this study. In our experiments, LPS treatment led to a marked decrease in viability and osteogenic differentiation, as well as an increase in inflammatory response in PDLSCs, suggesting that the periodontitis model in vitro was successfully induced.

SIRT6 is a key regulator of aging and has a certain influence on the differentiation of embryonic stem cells (ESC) [27]. The impact of SIRT6 on PDLSCs, however, has not been looked into. Lee et al. showed that SIRT6 expression in osteoblasts was significantly inhibited in induced apical periodontitis [28]. Consistent with this finding, in our experiment, it can be seen apparently that the expression of SIRT6 in LPS-induced PDLSCs showed a downward trend. It has been found that the loss of SIRT6 in mouse bone marrow cells inhibited bone mineral density and osteogenic differentiation of these cells [29]. When SIRT6 was knocked down, bone marrow mesenchymal stem cells showed decreased bone differentiation and osteogenic ability [30]. A previous study showed that root development and tooth eruption of mandibular first molars was delayed in SIRT6 knockout mice [31]. Moreover, the overexpression of SIRT6 could inhibit pulpitis by activating the TRPV1 channel [32]. Furthermore, SIRT6 has been shown to protect the osteogenic transdifferentiation of vascular smooth muscle cells through RUNX2 in chronic kidney disease [33]. SIRT6 promotes osteogenic differentiation of mesenchymal stem cells by regulating BMP signaling [34]. Therefore, we constructed SIRT6 overexpression plasmid and found that SIRT6 overexpression could prevent LPS-induced loss of viability, inflammatory response, and promote the expression of osteogenic related proteins BMP2 and RUNX2, thus promoting the ability of osteogenic differentiation. However, Huang et al. proposed that the down-regulation of SIRT6 promoted cementum formation [12]. This is somewhat different from our results, but the difference between our paper and this one lies in the different cells we studied. PDLSCs were used in our paper, and the effect of SIRT6 on the differentiation of cementoblast cells was discussed in Huang’s paper. We will further explore the differences between the two in future experiments.

Association of KLF5 to SIRT6 promoter was predicted by the JASPAR website. We then verified the binding ability between KLF5 and SIRT6 promoter through luciferase reporters and ChIP assays. Additionally, the finding that the transcription factor KLF5 positively regulated SIRT6 was also confirmed. During tooth development in mice, KLF5 mediated odontoblast differentiation by regulating the expression of dentin-specific extracellular matrix gene [35,36]. In addition, KLF5 acted as a key player in the osteogenic differentiation of human periodontal membrane cells [15]. In addition, KLF5 has been shown to mediate RUNX2 induction and play a key role in high phosphate induced vascular smooth muscle calcification [37]. Through our investigation, it was found that the interference of KLF5 could reverse the promoting effect of SIRT6 on LPS-induced PDLSCs proliferation and osteogenic differentiation and reverse the inhibitory effect of SIRT6 on LPS-induced PDLSCs inflammatory response.

It is widely considered that dysregulation of NF-κB signaling is responsible for the progression of human diseases [22]. Of note, NF-κB pathway is instrumental in the development of periodontitis. For instance, osteoclast formation could be affected by regulation of NF-κB pathway [38]. TNF-α-induced osteogenic injury of human periodontal membrane stem cells was reversed by inhibiting the NF-κB/NLRP3 inflammatory pathway [39]. The low expression of SIRT6 was closely associated with activation of NF-κB pathway in LPS-treated human dental pulp cells [32]. In our experiments, we found that the expression of NF-κB pathway-related proteins was enhanced following the LPS treatment, and KLF5-mediated SIRT6 inhibited the NF-κB pathway. In future experiments, our experimental findings need to be further verified by adding pathway inhibitors or activators.

## Conclusion

We found that KLF5-mediated SIRT6 promoted osteogenic differentiation and inhibited inflammatory injury of LPS-induced PDLSCs by inhibiting NF-κB pathway. Our study provides a new direction for the investigation into the pathogenesis of periodontitis and provides a theoretical basis for the treatment of periodontitis.

## Research highlights

- Transcription factor KLF5 stimulates the up-regulation of SIRT6
- SIRT6 promotes osteogenic differentiation of PDLSCs during periodontitis via NF-κB pathway
- SIRT6 inhibits inflammatory injury of PDLSCs during periodontitis via NF-κB pathway
